# Intervention Scalability Assessment Tool: A decision support tool for health policy makers and implementers

**DOI:** 10.1186/s12961-019-0494-2

**Published:** 2020-01-03

**Authors:** Andrew Milat, Karen Lee, Kathleen Conte, Anne Grunseit, Luke Wolfenden, Femke van Nassau, Neil Orr, Padmaja Sreeram, Adrian Bauman

**Affiliations:** 1 Centre for Epidemiology and Evidence, New South Wales Ministry of Health, 100 Christie Street, St Leornards, North Sydney, NSW 2065 Australia; 2 The Australian Prevention Partnership Centre, 10 Jones Street, Ultimo, NSW 2065 Australia; 3 School of Public Health, University of Sydney, Camperdown, NSW 2050 Australia; 4 University of Newcastle, Callaghan, NSW 2308 Australia; 5 Department of Public and Occupational Health, Amsterdam UMC, Vrije Universiteit Amsterdam, Amsterdam Public Health Research Institute, Amsterdam, The Netherlands; 6 The Poche Centre for Indigenous Health, University of Sydney, Camperdown, NSW 2050 Australia; 7 McMaster Health Forum, McMaster University, 1280 Main St W, Hamilton, ON L8S 4L8 Canada

**Keywords:** Implementation, scale-up, assessment support tool, scalability

## Abstract

**Background:**

Promising health interventions tested in pilot studies will only achieve population-wide impact if they are implemented at scale across communities and health systems. Scaling up effective health interventions is vital as not doing so denies the community the most effective services and programmes. However, there remains a paucity of practical tools to assess the suitability of health interventions for scale-up. The Intervention Scalability Assessment Tool (ISAT) was developed to support policy-makers and practitioners to make systematic assessments of the suitability of health interventions for scale-up.

**Methods:**

The ISAT was developed over three stages; the first stage involved a literature review to identify similar tools and frameworks that could be used to guide scalability assessments, and expert input to develop draft ISAT content. In the second stage, the draft ISAT tool was tested with end users. The third stage involved revising and re-testing the ISAT with end users to further refine the language and structure of the final ISAT.

**Results:**

A variety of information and sources of evidence should be used to complete the ISAT. The ISAT consists of three parts. Part A: ‘setting the scene’ requires consideration of the context in which the intervention is being considered for scale-up and consists of five domains, as follows: (1) the problem; (2) the intervention; (3) strategic/political context; (4) evidence of effectiveness; and (5) intervention costs and benefits. Part B asks users to assess the potential implementation and scale-up requirements within five domains, namely (1) fidelity and adaptation; (2) reach and acceptability; (3) delivery setting and workforce; (4) implementation infrastructure; and (5) sustainability. Part C generates a graphical representation of the strengths and weaknesses of the readiness of the proposed intervention for scale-up. Users are also prompted for a recommendation as to whether the intervention (1) is recommended for scale-up, (2) is promising but needs further information before scaling up, or (3) does not yet merit scale-up.

**Conclusion:**

The ISAT fills an important gap in applied scalability assessment and can become a critical decision support tool for policy-makers and practitioners when selecting health interventions for scale-up. Although the ISAT is designed to be a health policy and practitioner tool, it can also be used by researchers in the design of research to fill important evidence gaps.

## Background

In order to achieve population-wide benefits and foster sustainable policy and programme development, health interventions found effective within controlled or research settings should be scaled up [1, 2]. Here, we refer to the process of ‘scale-up’ or ‘scaling up’ as “*deliberate efforts to increase the impact of successfully tested health interventions so as to benefit more people and to foster policy and program development on a lasting basis*” [2]. However, there have been few documented examples of efficacious population health interventions being scaled up successfully in developed countries [3–6]. A contributing factor is the lack of pragmatic studies demonstrating how pilot interventions can be disseminated in real-world settings, as research has predominantly focused on describing disease risk patterns [7] or intervention efficacy testing, rather than disseminating interventions across systems [5, 8, 9]. Other factors include lack of knowledge, skills and capacity among policy-makers and practitioners to determine the suitability of interventions for scale-up [10], and the likelihood that political and resourcing factors are often more powerful influences on scale-up decisions than whether interventions are evidence based [8]. Hence, it is important to provide better support to policy-makers and practitioners to more readily assess the suitability of interventions for scale-up and their scalability within a specific context.

In this paper, we define scalability as *“the ability of a health intervention shown to be efficacious on a small scale and/or under controlled conditions to be expanded under real world conditions to reach a greater proportion of the eligible population while retaining effectiveness …*” [1]. Assessing scalability has been identified as a fundamental step in any scaling up process [2, 11–13], as it helps to avoid unnecessary expenditure of resources and efforts to scale up unsuitable interventions [14]. Furthermore, assessing scalability generally requires an assessment of a range of considerations, including feasibility, acceptability, costs, sustainability and, most importantly, adaptability (i.e. to suit the needs of the context in which it is to be scaled up), which are often difficult to assess [1]. While a growing number of frameworks and guides offer step-by-step processes for scaling up evidence-based interventions and identifying factors, including scalability, that should be considered throughout the scaling up process [15–17], these guides do not offer practical tools that policy-makers can use to conduct structured scalability assessments. Where scalability tools do exist, such as in Cooley et al.’s [11, 18] Scaling Up Management Framework, the scalability checklist is brief and does not provide a mechanism for evidence gathering or a process for the comprehensive and systematic assessment of scalability. Moreover, the Scaling Up Management Framework checklist was developed primarily for use in low- to middle-income country (LMIC) contexts and thus may have less utility in high-income countries.

The aim of this study was to develop an Intervention Scalability Assessment Tool (ISAT) that enables policy-makers and practitioners to make systematic assessments of the suitability of health interventions for population scale-up within high-income country health and community settings.

## Methods

**Fig. 1:**
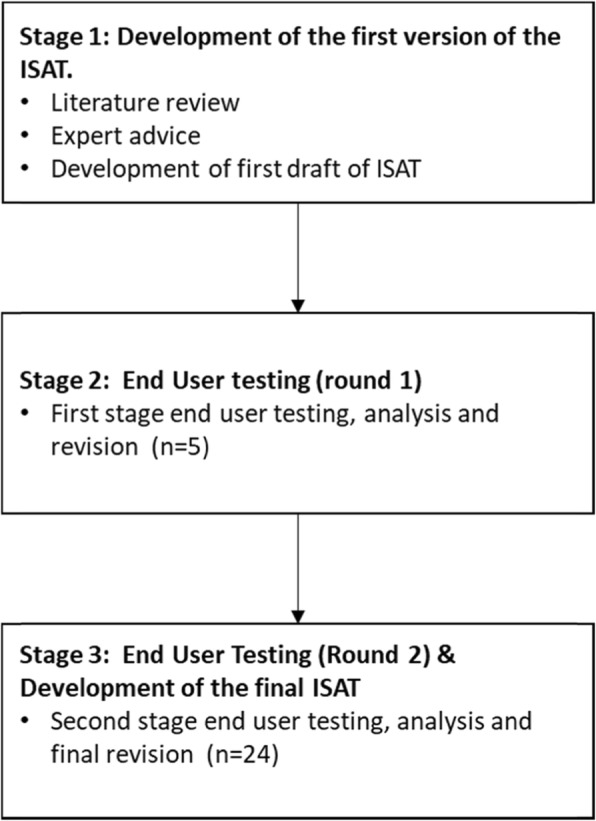
Summary of the research process

The ISAT was developed in three stages; the first stage involved a literature review and expert consultation to identify existing scalability tools and the potential domains to be covered by the initial version of the ISAT. The second stage involved testing the initial version of the ISAT, with five end users and developing a second draft based on respondent feedback. The third stage involved testing the second version with 24 end users and using feedback to develop the final ISAT. Figure 1 summarises the key steps in the development of the ISAT. More detail on the methods employed in each stage follows.

### Stage 1: Development of the first version of the ISAT

A literature review was conducted to identify frameworks, guides, checklists or tools associated with the scalability or scaling up of health interventions that could be used to inform the development of the ISAT. The review was conducted in two phases. In Phase 1, a keyword search of studies published in English between 1990 and 2017 was performed using MEDLINE and SCOPUS. The search terms were (“scalability” OR “scale up” OR “upscale” OR “up-scale”) AND (“framework” OR “guide” OR “checklist” OR “tool” OR “readiness” OR “assessment”).

The logic behind including scale-up frameworks in the review was that ‘scalability assessment’ is an initial step in a number of such frameworks [1, 11, 13]. The search strategy was developed by AM and abstracts were retrieved and assessed for relevance to the study by KL. In Phase 2, among abstracts deemed relevant in Phase 1, full papers were retrieved and assessed against the following review inclusion criteria:

- Peer reviewed
- Published in English from 1990 to 2017
- Described frameworks, guides, checklists or tools associated with scalability considerations or scale-up of health interventions

For the purposes of the review, ‘frameworks or guides/guidelines’ were included if they provided structured approaches or step-by-step guides by which scale-up or scalability could be organised or assessed. ‘Checklists’, ‘tools’ or ‘assessments’ were included if they provided a list of features or considerations with respect to making decisions regarding scalability or scale-up.

Papers were excluded if they described:

- The concept of scalability without providing a framework or checklist
- Scale-up of information technology systems not related to health interventions
- Scale-up and evaluation of specific interventions
- Study protocols for potential scale-up
- Medical testing procedures
- Statistical modelling without frameworks, guidelines or tools to assist decision-makers to assess the scalability of health interventions
- Facilitators and/or barriers to scale-up within specific interventions or general experiences of scale up that did not provide a framework or checklist for guidance
- New scale-up concepts that did not include guidance on how they were to be applied

To supplement the peer-reviewed scientific literature, an open keyword search in the Google search engine was also conducted using the same variations of the above search terms. As recommended by the Canadian Agency for Drugs and Technologies in Health [20] for systematic searches of grey literature, the top 50 hits were reviewed. The reference lists of all included papers were further scanned for potentially relevant tools or articles.

Finally, a convenience sample of Australian experts (*n* = 3) in scaling up health interventions were also consulted to identify sources in the grey and peer-reviewed literature of potential relevance to the task of developing the draft ISAT. Experts were selected for their knowledge of the scale-up literature, implementation frameworks and experience with scale-up processes.

The relevant information from the results of the peer-reviewed and grey literature review was extracted and integrated to map existing frameworks, guides, checklists and tools used for scaling up health interventions and scalability assessment across key characteristics, as shown in Table 1. Table 1 describes the terms and categories used to distinguish the features between the frameworks, guides, checklists and tools outlined in Table 3.

**Table 1:** Definitions of categories

Category	Variable definition	Additional definitions
Model/Framework	Model/Framework name	Where there is no specific name, a generic descriptor was used
Stage of scale-up	Focus on which part of the process	• Pre-scale-up: steps or activities undertaken prior to embarking on the scale-up of an evidence-based intervention • Scale-up: steps and/or activities required to ‘disseminate’ the evidence-based intervention • Implementation: the process of using or integrating the evidence-based intervention within a setting [21]
Focus area	Focus on disease/condition type	• Non-specific/generalisable: the framework/model/checklist could be applied to several different disease contexts, even though it may have been developed using a specific disease frame • Disease/condition specific: the framework/model/checklist has been designed to be applied to the scale-up of interventions that are specific to a particular disease/condition
Key components	Description of the key components of the model/framework	
Process of development	Description of the key methods undertaken to develop the model/framework	• Literature review • Delphi process • Qualitative research (including interviews) • Case study
Context	Description of the context from which the model/framework was derived	• High-income country • Global health/low- and middle-income country

AM produced an initial draft of the ISAT in light of the literature and expert opinion. The domains identified in this initial draft were drawn from those described in existing scalability checklists and scale-up frameworks. This initial draft was then circulated among the study investigators for over several rounds and their feedback was incorporated into the draft of the ISAT tested in the first round of end-user interviews.

### Stage 2: End-user interviews (Round 1) and testing

To test the draft versions of the ISAT, interviews were conducted with a range of Australian policy-makers, implementation science academics and clinicians (hereafter known as ‘end users’) with experience in scaling up health interventions. Relevant end users with experience in scaling up public health interventions were initially identified by study investigators and followed by a passive snowballing recruitment method to obtain additional informants. Interviews with policy-makers and practitioners were conducted over two rounds; the first round was conducted between December 2017 and January 2018 and, following further revisions to the ISAT, a second round was conducted between May and September 2018. A total of 34 end users were invited to participate in the process, from which 29 interviews were conducted (85% response rate). The remaining end users (*n* = 5) were not able to be contacted or did not respond. The majority of interviews (*n* = 25) were conducted over the telephone, and a minority (*n* = 4) were conducted face to face. Different end users participated in each round of interviews.

For both interview rounds, topic guides were developed and questions were generated with a view to understanding the processes that policy-makers use to make decisions on scaling up health interventions and the difficulties encountered in that process as well as to obtain feedback on the content and structure of ISAT drafts. Ethics approval was obtained from the University of Sydney’s Human Research Ethics Committee (2017/828). A summary of the topics covered in the two rounds of end-user testing can be found in Table 2 and the full interview guides are provided in Additional file 1.

**Table 2:** Summary of topics covering end-user interview guides

Topic	Description
Professional background	Obtaining a description of the end users’ professional background and experience on scaling up population health interventions
Respondent’s experience in the decision-making process to scale-up a population health intervention	Developing an understanding of a specific intervention(s) that had been scaled up, which included a description of the problem being addressed, the context in which it was scaled up as well as the process of scaling up
General reflection on the process of scaling up interventions	Ascertaining information pertaining to the key elements in the process of scaling up such as identifying the key actors and their role, the role of evidence in decision-making and the key influences in the decision-making process
Feedback on the ISAT	Eliciting general perceptions of the tool, including the perceived purpose and potential users of the tool, the design and content of the tool, and the perceived applicability of the tool, along with the language and presentation of the tool

KL and KC conducted interviews, with KL acting as the lead interviewer. The interviews were audio recorded and transcribed verbatim by an external transcription service (www.rev.com). All end users were invited to participate via email and provided their verbal and signed consent. All interview transcripts were de-identified for the purpose of analysis and reporting. Thematic analysis [32] was used to identify themes across the interview responses and were used to reshape each iteration of the ISAT. The analysis was conducted by KL and findings were discussed with all members of the study investigative team and used as part of the revision process. Between each round of interviews, the responses obtained from end users were collated and thematically coded and used to revise the ISAT.

### Stage 3: End-user interviews (Round 2) and development of the final ISAT

The last stage of the process included the thematic analysis and coding of end-user responses from the second round of interviews to produce the final ISAT.

## Results

### Literature review

The initial search in MEDLINE and SCOPUS databases with the keywords and keyword combinations yielded 2769 abstracts and searches of the grey literature identified a further 4 documents, making a total of 2773 documents. Following exclusion of duplicate records (*n* = 134), of the 2639 paper abstracts and documents reviewed for relevance against the review criteria, 2554 were excluded.

**Fig. 2:**
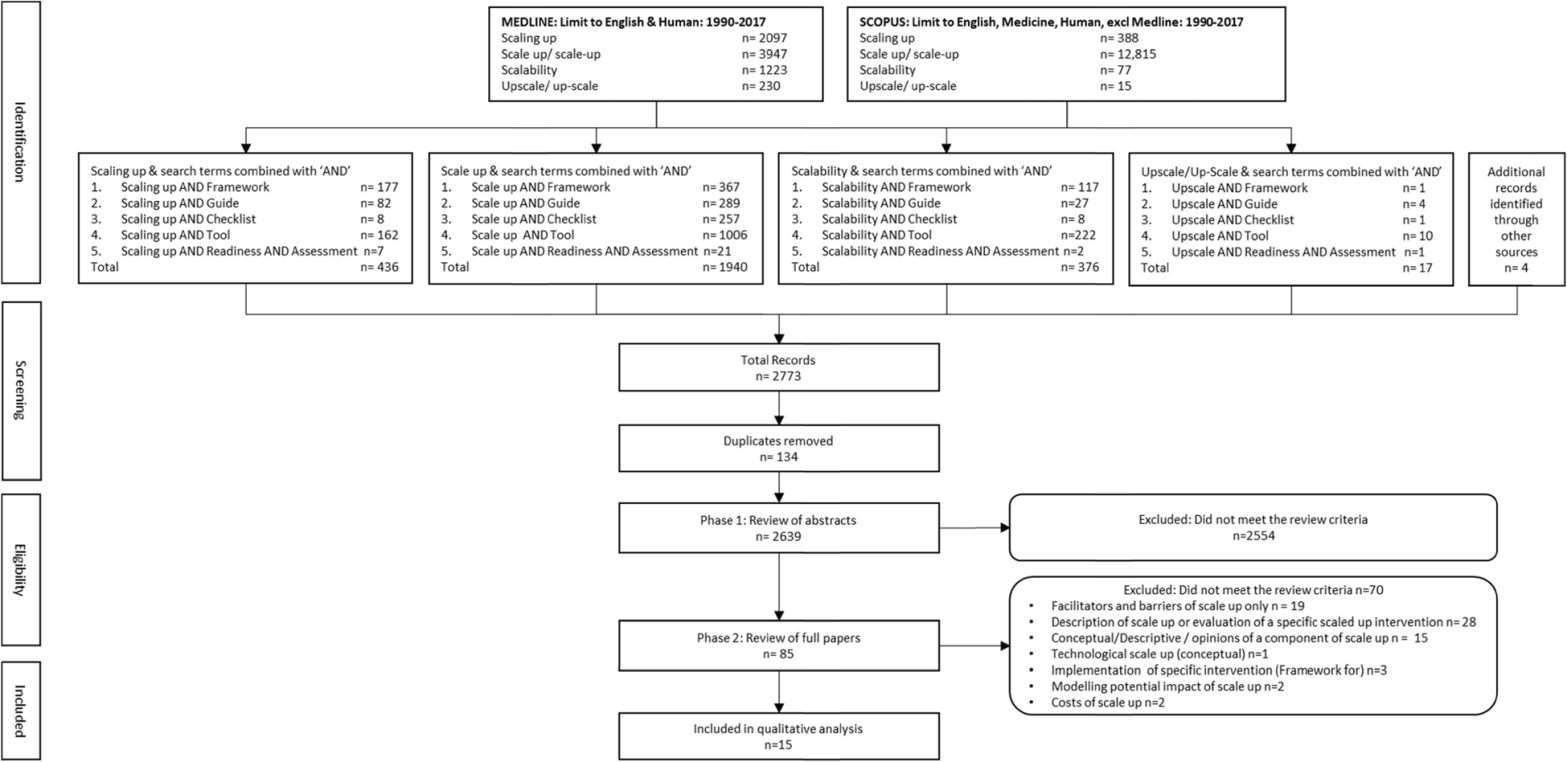
Literature search PRISMA flow chart

The full text versions of remaining papers and reports (*n* = 85) were retrieved and reviewed against the inclusion criteria for Phase 2 (Fig. 2). A total of 28 papers described scaled up interventions and/or evaluations of scaled up interventions. A further 19 papers provided insights into facilitators and barriers to scale-up, but generally specific to their intervention and/or context. There were a small number of papers either generating new scale-up concepts or providing opinions on scale-up in general (*n* = 15). Finally, 15 papers/reports were noted as describing scale-up frameworks, tools and checklists, and were included in the final review. The literature search is reported according to the Preferred Reporting Items for Systematic review and Meta-Analysis (PRISMA) statement (www.prisma-statement.org.au).

### Characteristics of existing frameworks, guides, checklists and tools used to scale-up health interventions

The literature review revealed a number of existing frameworks, guides, checklists and tools (hereafter known as ‘frameworks’) to guide the scaling up of health interventions (Table 3). Eleven of the 15 frameworks arose out of (or were developed specifically for) the global health and/or LMIC context. These articles had a primary focus on communicable diseases such as HIV/AIDS or general LMIC health promotion programmes, including maternal and child health improvement programmes. Similarly, 70 abstracts were excluded following the full text review Phase 2 as they only provided information on facilitators, barriers and case studies of successfully scaled up programmes from the global health or LMIC contexts.

**Table 3:** Existing frameworks, checklists and tools used for scaling up health interventions and scalability assessment

Model/framework	Stage of scale-up	Focus area	Key components	Process of development	Context	Reference
Scalability considerations	Pre-scale-up	Non-specific/generalisable	Key features of scalability of an intervention should consider: 1. Effectiveness 2. Reach and adoption 3. Human, technical and organisational resources 4. Costs 5. Intervention delivery 6. Contextual factors 7. Appropriate evaluation approaches	Literature review/expert Delphi process	High-income country	Milat et al. 2012 [1]
Scalability considerations	Pre-scale-up	Maternal and newborn	Key attributes of scalable health innovations: 1. Relevant and important – addresses a health need 2. Effective and advantageous – impacts positively on health and is advantageous over other innovations 3. Observable benefits – benefits and health impacts are visible 4. Acceptable to health workers and communities – culturally acceptable, works with existing community structures 5. Simple and low cost – low cost to implement at scale 6. Aligned and harmonised – builds on existing government health systems 7. Adaptable – adaptable to different contexts 8. Sustainable – low recurrent costs or local income-generating schemes	Qualitative study (interviews)	Global health/low- and middle-income country (LMIC)	Spicer et al. 2014 [22]
Scaling Up Management Framework (SUM) Scalability Checklist	Pre-scale-up	Non-specific/generalisable	Seven key categories when considering scalability: 1. How convincing is the scaling strategy? 2. Is the intervention credible 3. How strong is the support for change? 4. Does the model have relative advantage over existing practices? 5. How easy is the model to transfer and adopt? 6. How good is the fit between the intervention and adopting organisation? 7. Is there a sustainable source of funding?	Literature and previous framework development	Global health/LMIC	Cooley et al. 2016 [18]
Taking innovations to scale – scalability checklist	Pre-scale-up/scalability checklist	International Fund for Agricultural Development	Seven key categories when considering scalability: 1. Is the model credible? 2. How observable are the model’s results? 3. How relevant is the model? 4. Does the model have relative advantage over existing practices? 5. How good is the fit between the intervention and adopting organisation? 6. How testable is the model? 7. Is there a sustainable source of funding?	Literature and case studies	Global health/LMIC	Cooley & Linn 2014 [11]
Scaling up process	Scale-up process	Maternal and newborn	Key activities required to catalyse scale-up: 1. Designing innovations for scale 2. Integrating scale-up within programme plans 3. Building organisational capacity 4. Advocating effectively with government decision-makers 5. Generating and communicating strong evidence 6. Ensuring government involvement throughout the project 7. Invoking policy champions and network of allies 8. Aligning with government systems, policies, priorities and targets 9. Harmonising efforts with other development partners and implementers 10. Supporting and building the capacity of government for scale-up 11. Working with community leaders, media and others to stimulate diffusion of innovations among communities	Qualitative study (interviews)	Global health/LMIC	Spicer et al. 2014 [22]
Framework for scaling up health interventions	Scale-up process	Non-specific/generalisable	Framework for scaling up health interventions. Describes four key steps: 1. Set-up, to prepare the ground for introduction and testing of the intervention that will be taken to full scale 2. Develop the Scalable Unit, i.e. an early testing phase 3. Test of scale-up, to test the intervention in a variety of settings that are likely to represent different contexts that will be encountered at full scale 4. Go to full scale, to rapidly enable a larger number of sites or divisions to adopt and/or replicate the intervention	Literature review/Case study	Global health/LMIC	Barker et al. 2016 [23]
Scale-up of exclusive breastfeeding	Scale-up process	Health Promotion/Maternal and Child nutrition	Outlined key steps for scaling up maternal breastfeeding programmes 1. Assess situation – create a policy environment 2. Define roles, relationships and responsibilities of all partners and establish agreements 3. Review technical support 4. Define programme strategy 5. Mobilise resources 6. Provide training and technical assistance 7. Develop and use monitoring and evaluation systems 8. Monitor coverage and quality 9. Measure impact and cost 10. Provide novel approaches for testing and continuing innovation	Literature review	Global health/LMIC	Bhandari et al. 2008 [19]
Schemata for considering context in scale-up	Scale-up process	HIV	Presents schemata for progression from efficacy to full scale implementation on three dimensions considering contextual elements using HIV as a case study. The three dimensions include: 1. Determinants and their pathways 2. Framing the research question 3. The design of the intervention contrasting between contextualised vs. standardised interventions	Literature review/Case study	Global health/LMIC	Edwards & Barker [24]
Scale-up framework	Scale-up process	HIV	Framework of 10 key domains critical to successful scale-up: 1. Fiscal support 2. Political support 3. Community involvement 4. Partnerships 5. Balancing flexibility/adaptability and standardisation 6. Supportive policy, regulator and legal environment 7. Building and sustaining strong organisational capacity 8. Transferring ownership 9. De-centralisation 10. Ongoing focus on sustainability	Literature review/case study	Global health/LMIC	Hirschhorn et al. [25]
Program Assessment Guide – how to make decisions relating to design, implementation and scale-up	Scale-up process	Health promotion/nutritional programmes	Program Assessment Guide – designed as a structured, systematic method for countries to make decisions related to design, implementation and scale-up. Nine key steps across three categories. Category 1: Laying the groundwork • Step 1 – Clarifying the problem and proposed solution • Step 2 – Setting vision and goals • Step 3 – Reaching the most vulnerable • Step 4 – Delivery system, mapping out the system • Step 5 – Identifying people, roles and responsibilities Category 2: Building or strengthening the programme • Step 6 – meeting the needs • Step 7 – action planning, including timeframes Category 3: Strengthening the decision support and the enabling environment, which also includes: • Step 8 – monitoring, evaluation and quality improvements • Step 9 – organising, leading and managing the follow through	Case studies	Global health/LMIC	Pelletier et al. [26]
Nine steps to scaling up – WHO ExpandNet	Scale-up process	Non-specific/generalisable	ExpandNet framework involves nine steps: 1. Planning actions to increase the scalability of the innovation 2. Increasing the capacity of the user organisation to implement 3. Assessing the environment and planning actions to increase the potential for success 4. Increasing the capacity of the resource team to support scaling up 5. Making strategic choices to support vertical scaling up 6. Making strategic choices to support horizontal scaling up 7. Determining the role of diversification 8. Planning actions to address spontaneous scaling up 9. Finalising the scaling up strategy and identifying next steps	Literature and qualitative research	Global health/LMIC	World Health Organization ExpandNet [27]
Scaling up management framework (SUM)	Scale-up process	Non-specific/generalisable	Includes three key steps: Step 1: developing a scaling up plan, including assessing for scalability Step 2: establishing pre-conditions for scaling up Step 3: implementing the scaling up process based on the identification of factors that can promote extension and sustainability Same as Cooley & Linn [11], seven key categories when considering scalability: 1.Is the model credible? 2.How observable are the model’s results? 3.How relevant is the model? 4.Does the model have relative advantage over existing practices? 5.How good is the fit between the intervention and adopting organisation? 6.How testable is the model? 7.Is there a sustainable source of funding?	Literature and qualitative research	Global health/LMIC	Cooley et al. 2016 [18]
Scaling up global health interventions: framework for success	Scaling up framework	Non-specific/generalisable	Description of six components core to the scaling up process: 1. Attributes of specific tool or service being scaled up 2. Attributes of the implementers 3. Chosen delivery strategy 4. Attributes of the adopting community 5. Socio-political context 6. Research context	Literature review, interviews	Global health/LMIC	Yamey [28]
Scaling up population health interventions	Pre-scale-up and scaling up population health interventions	Non-specific/generalisable	Description of a four-step process for scaling up interventions: 1. Scalability assessment to assess the suitability of the intervention for scaling up 2. Develop a scaling up plan – create a vision of what scaling up will look like and a compelling case for action 3. Prepare for scaling up – securing resources and building a foundation of legitimacy and support for the scaling up plan 4. Scale-up – the main tasks that should be addressed during scale-up	Literature review and expert Delphi process	High-income country	NSW Ministry of Health [13]
Readiness assessment – I-RREACH Tool	Implementation planning	Cardiovascular disease	Three stages of activity as part of the process to assess for implementation readiness, I-RREACH tool • Stage 1 – Building a community profile • Stage 2 – Gathering information on key stakeholder’s perspectives • Stage 3 – Gathering information on community perspectives There are eight key information domains: 1. Basic community descriptions 2. Leadership 3. Community programmes 4. Local understanding of the health issue 5. Resources and planning 6. Perceived fit of the intervention with community objectives 7. Infrastructure and technology 8. Readiness for community-based research	Community-based participatory research methodology and qualitative study (interviews)	High-income country and LMIC	Maar et al. 2015 [29]
Implementation rules for scale-up	Implementation	Mental health	Describes five implementation rules for consideration when planning for the scale-up of mental health services: 1. Assess context 2. Identify priority care pathways and map them across skill needs 3. Specify decision supports, supervision and triage rules 4. Apply and use quality improvement practices 5. Plan for sustainability and capacity-building	Case studies	Global health/LMIC	Belkin et al. [30]
Five-step framework scaling up strategy of the European Partnership on Active and Healthy Ageing	Scaling up framework	Healthy ageing/chronic respiratory diseases	One of the priority areas of the strategy is to scale-up and replicate successful innovative integrated care models for chronic respiratory diseases. There is a five-step framework for developing scaling up strategies: What to scale up 1. Step 1: database of good practices 2. Step 2: assessment of viability of the scaling up of good practices 3. Step 3: classification of good practices for local replication How to scale up 4. Step 4: facilitating partnerships for scaling up 5. Step 5: implementation key success factors There were also steps for individual services planning to scale up: 1. Planning and initiating the service 2. Setting up the system for change 3. Organisational process and design choices 4. Appropriate resourcing for equipment 5. Integration of clinical record systems 6. Creating capacity Monitoring, evaluation and dissemination	Multi agency/multinational partnership	High-income country	Bousquet et al. 2016 [31]

All of the frameworks and scaling up processes were developed using existing literature (theories and frameworks) and/or qualitative interviews with those who were part of a scale-up process or case studies. Of the 15 individual papers shown in Table 3, nine were primarily focussed on providing a framework for the scale-up process (i.e. dissemination), while two were focused on implementation. The remaining four either focused on what we have termed pre-scale-up (i.e. scalability considerations exclusively [1]) or as an important part of the scale-up process [11, 18, 22].

Of the ten frameworks and checklists providing steps for scale-up, five [1, 11, 13, 18, 22] specifically mentioned the assessment of scalability although guidance on assessing scalability was only provided by one author through two iterations [11, 18] of a scalability checklist (Table 3). This one-page checklist (called the ‘Scaling Up Management Framework Scalability checklist’) described seven categories for scalability and, within those categories, provided a series of questions to facilitate a decision of scalability across a three-point scale; users were not prompted to provide additional supporting text or evidence. The remaining three papers described scalability [1, 13, 22] and outline some of the key factors for consideration but only provided minimal or no guidance on how to perform a scalability assessment.

Table 3 summarises existing frameworks, checklists and tools used for scaling up health interventions and scalability assessment.

Table 4 describes key scalability concepts covered in existing scalability frameworks and checklists. Concepts covered in all of the existing scalability frameworks and checklists identified in the review included contextual considerations, evidence of effectiveness, scale-up and implementation considerations, workforce considerations, and costs of scale-up. Other common scalability concepts identified included the importance of problem definition, intervention adaptability and delivery system considerations. Only three of the five scalability frameworks and checklists covered the concept of intervention reach and acceptability.

**Table 4:** Scalability concepts covered in existing scalability frameworks and checklists

Scalability considerations	Milat et al. 2012 [1]	Cooley et al. 2014 [11]	Cooley et al. 2016 [18]	MoH NSW et al. 2014 [13]	Spicer et al. 2014 [22]
Problem definition		√	√	√	√
Contextual considerations	√	√	√	√	√
Comparison against similar interventions		√	√	√	√
Evidence of effectiveness	√	√	√	√	√
Intervention reach and acceptability	√			√	Acceptability only
General scale-up and implementation considerations	√	√	√	√	√
Workforce considerations	√	√	√	√	√
Delivery system considerations	√	√	√	√	
Costs of scale-up	√	√	√	√	√
Intervention adaptability	√	√	√	√	
Monitoring and evaluation	√				
Sustainability		√	√		√

Table 5 summarises the key functions of the existing scalability frameworks and checklists. Of note, only two of the five existing scalability frameworks or tools provided structured scalability assessments [11, 18] and none of the assessment tools provided a process for evidence gathering across different scalability domains.

**Table 5:** Key functions of existing scalability frameworks and checklists

Reference	Proposes scalability concepts	Poses scalability questions for consideration	Structured scalability assessment	Provides a summative assessment	Process for evidence gathering across scalability concepts
Milat et al. 2012 [1]	√	×	×	×	×
Cooley et al. 2014 [11]	√	√	√ (3-point scale)	√	×
Cooley et al. 2016 [18]	√	√	√ (3-point scale)	√	×
MoH NSW et al. 2014 [13]	√	√	×	×	×
Spicer et al. 2014 [22]	√	×	×	×	×

**Table 6:** Characteristics of the end users interviewed

Role	Total (*n* = 29)	Organisation type	Total (*n* = 29)
Senior executive	4	State/local-based health organisation	21
Mid-level policy-maker	15	Non-government organisation	2
Junior policy-maker	6	Academic institution	4
Academic/Clinician	4	Statutory body	2

### Thematic analysis of interviews with end users

#### Respondent characteristics and scaling up experience

The end users interviewed (*n* = 29) were from all Australian states and territories and represented a number of different organisation types and roles (Tables 6, 4). All indicated that they had personally either been a part of, or had led, the scale-up of health interventions.

End users mainly identified as mid-level policy-makers and came from state and/or local-based health organisations. Those identifying as Senior Executives indicated that they had been and/or are currently part of the decision-making capacity within their organisations on funding and scale-up of interventions. Data from interviews with respondents are described below under the themes of importance of decision support tools, when and how to use the ISAT, perceptions on the likely process required to complete the ISAT, additions to the ISAT and potential limitations. This thematic analysis informed the development of ISAT and is followed by a description of the final ISAT.

#### Importance of decision support tools for policy and practice

The concept of an assessment tool that aims to facilitate structured thinking and consideration of potential implementation issues when scaling up promising interventions was widely supported amongst interviewed end users. From a decision-maker perspective, it was reported that having well thought-out considerations generated by completing the ISAT would prompt more detailed discussion of the challenges that might be faced when implementing an intervention at scale and, by doing so, would help improve implementation-related decision-making.

“*I think this is a tool that would help people to craft well thought through arguments as to why they think a scaled up version of a program would be of benefit, and the kinds of impacts and investment it … Well, the investment that you’d need and the delivery system that you’d need, and then the kind of impacts that you’d see at a population level, they’d be the sorts of things that then become important in even considering an idea.*” (P04)

Others expressed that a tool like the ISAT could be included as part of funding application processes as it would add rigour, consistency and, potentially, accountability to funding assessment processes. Being able to standardise the application of the scalability assessment tool to funding decision-making processes would also be useful where there are competing claims on budgets with multiple interventions for consideration.

#### When/how to use a decision support tool like the ISAT

End-user interviewees reported that there was value in using the ISAT beyond assisting decision-making on whether to fund interventions for scale-up, and for informing decisions about interventions that are currently being scaled up or delivered at scale (i.e. whether to continue, expand, scale back or terminate these activities). Other potential uses identified for the ISAT were as a tool to advocate for further funding or support for a promising intervention, as part of the strategic planning process in determining promising interventions for further testing and/or potential scale-up, and as a mechanism for documenting information and processes of the intervention and its subsequent scale-up to capture key learnings for quality improvement activities.

### Perceptions on the likely process required to complete the ISAT

Despite not having piloted the ISAT on a real-life intervention, a number of end users suggested that the ISAT would be best completed as a group process since the relevant data and information for answering the questions is unlikely to be found in any one location or from one individual alone. Many indicated that having multiple viewpoints to inform the final assessment process would be valuable in facilitating a more balanced and comprehensive assessment.

#### Suggested additions to the ISAT

While not listed in detail in this paper, there were numerous suggestions for additions, exclusions and modifications to the content of the ISAT through both rounds of interviews. One of the key additions included the need for greater emphasis on the consideration of sustainability of interventions post scale-up. Respondents reported that, in their experience of scaling up, sustainability (particularly financial sustainability) was often not considered beyond the initial funding period. Using the ISAT in the planning stages where sustainability is addressed could help increase the likelihood of longer-term sustainability when it is implemented.

The overall comparison across the key domains of the tool was added to the tool following the initial round of user feedback. Respondents reported that the resultant summative visualisation of the strengths and weaknesses of interventions considered for scale-up, using a mix research evidence, expert opinion, practitioner knowledge and contextual information, was very useful for decision-making.

#### Potential limitations of the ISAT

A number of limitations of the ISAT were identified through the interviews with end users, the first of which was that, while the ISAT could provide guidance on the scalability of efficacious interventions, its ability to provide guidance for interventions where the evidence base is not as strong or yet to be tested was challenging for some. Examples of this included interventions that were still in a pilot testing stage.

Secondly, it was noted that, potentially, those with less experience with the concepts and requirements of scaling interventions might find completing the ISAT challenging. There is a certain level of implied knowledge and skillset in order to complete the ISAT, leading to the observation that the ISAT would be best completed as part of a group process where different views and expertise would contribute to a balanced and comprehensive assessment.

#### The ISAT

The ISAT (Additional file 2) consists of three key parts — Part A: setting the scene, Part B: intervention implementation planning, and Part C: summary of scalability assessment — as outlined below.

### Part A: setting the scene

The purpose of this section is to outline the context for which the intervention is being considered for scale-up and consists of five individual domains, as outlined in Table 7.

**Table 7:** ISAT: PART A domains and objectives

Domain	Description of the domain
A1: The problem	Considers the problem that is being addressed. The questions in this domain seek a description of the problem, who it affects, what it affects and how it is currently being addressed (if at all)
A2: The intervention	Description of the proposed programme/intervention to address the problem
A3: Strategic/political context	Strategic/political/environmental contextual factors that are potentially important influences on any intervention to be scaled up
A4: Evidence of effectiveness	Level of evidence available to support the scale-up of the proposed intervention, such as scientific literature and/or other known evaluations of the intervention
A5: Intervention costs and benefits	Consideration of the known costs of the intervention delivery as well as any quantifiable benefits This includes the results of any types of economic evaluation studies

### Part B: intervention implementation planning

Part B considers the potential implementation and scale-up requirements of the intervention within five domains. The questions are designed to promote early thinking about potential implementation issues that would contribute to an intervention’s potential scalability. The information generated through this section can provide the basis for a detailed scale-up or implementation plan (Table 8).

**Table 8:** ISAT: Part B domains and objectives

Domain	Description
B1: Fidelity and adaptation	Proposed changes to the intervention required for scale-up
B2: Reach and acceptability	The likely reach and acceptability of the intervention for the target population
B3: Delivery setting and workforce	Define the setting within which the intervention is delivered as well as the delivery workforce
B4: Implementation infrastructure	Implementation infrastructure is required for scale-up
B5: Sustainability	Longer-term outcomes of the scale-up and how, once scaled up, the intervention could be made sustainable over the medium to longer term

### Part C: summary of scalability assessment

**Fig. 3:**
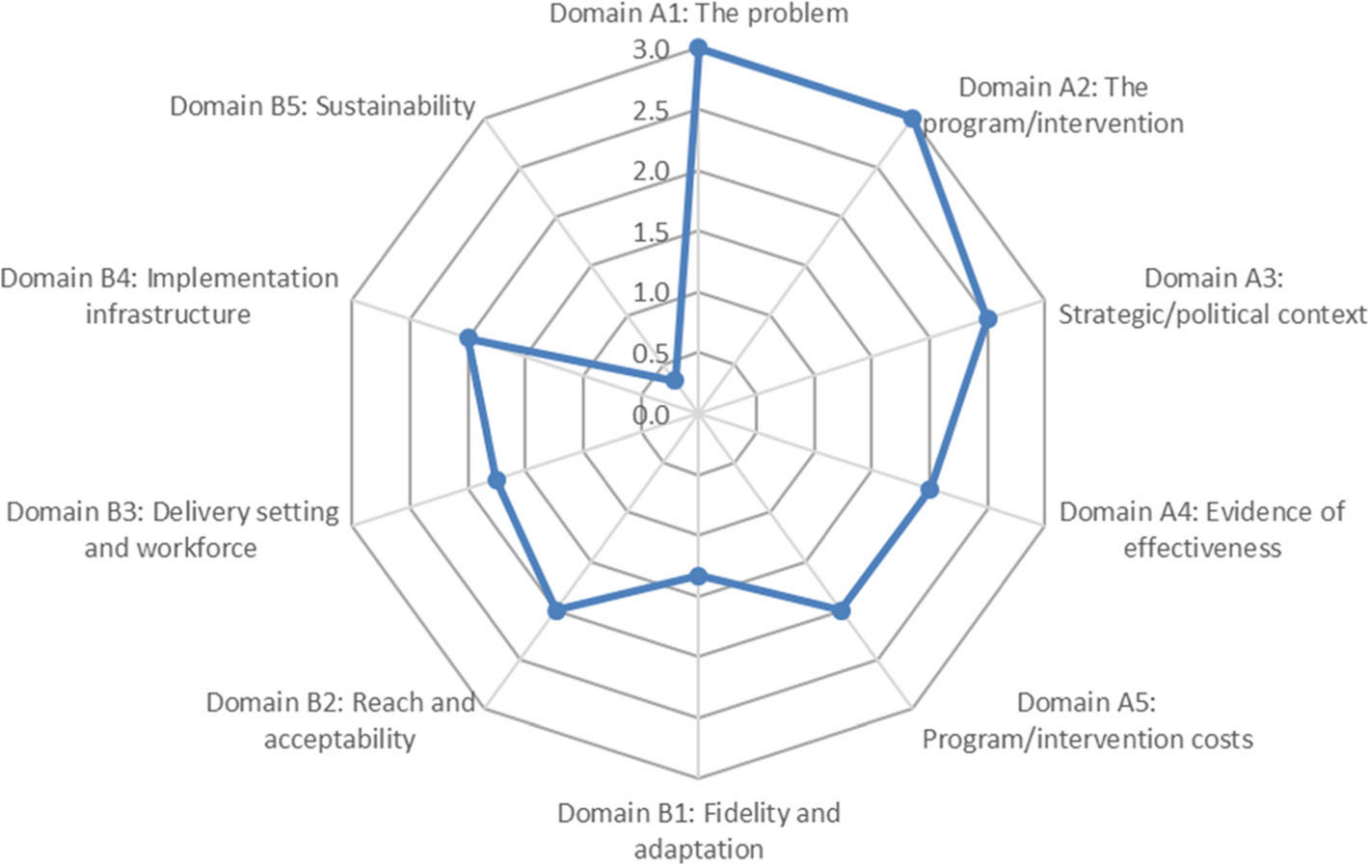
Example of ISAT spider web plot

At the end of each domain there are several questions, rated on a scale from 0 to 3, representing the readiness assessment for that domain. The purpose of these questions is to identify strengths and weaknesses for comparison across domains. Part C amalgamates the results from Parts A and B, including the summation of the assessment scores. A visual representation of the results of assessments for each domain in the form of a spider web plot may be generated to summarise the strengths and weaknesses of the proposed intervention in light of the scalability criteria and facilitate cross-domain comparisons (Fig. 3).

The final question prompts a recommendation about whether the intervention should be recommended for scale-up, whether the intervention is promising but likely to require further information or planning before scaling up, or whether it does not merit scale-up.

### Interpreting the ISAT findings

The questions in the ISAT are wide ranging and their intent is to stimulate thinking and promote the active consideration of factors that are important when assessing scalability. It is important to note that there is no ‘total’ or summary score that can be derived from combining the scores on the individual domains. The omission of such a total score is deliberate in recognition that not all the domains are necessarily equally important across all contexts and scenarios. Low scores in some domains may be acceptable in some situations, but not in others, depending on the perceived importance of the domain in the context of intervention of scale-up. Further, it may not be possible to answer all questions posed in the ISAT, but the absence of information is also informative. Visual representation of the scores across each domain serves to highlight areas in which evidence may need to be strengthened. It also provides a starting point through a structured process to facilitate discussions on the potential scalability and/or readiness of the intervention in question with a variety of decision-makers and other stakeholders.

## Discussion

While a variety of frameworks to guide scale-up processes exist in the literature, this review identified a major gap in the area of scalability assessment, particularly in high-income country contexts. It is worth noting that, while two scalability checklists were found in the literature [11, 18], both were from the same author and were simple, one-page checklists. These checklists ask users to tick responses to questions under different scalability domains but does not collect evidence under each domain to enable the graded assessment required for a comprehensive scalability assessment.

The three-part ISAT guides users through the known factors that affect the success of scaling up health interventions, encouraging evidence-based decision-making and reflection on resources required and potential threats to sustainability. The core domains contained in the ISAT were largely derived from the scale-up literature and refined based on feedback through interviews with end users. Key domains in Part A, such as defining the problem, intervention characteristics and contextual factors, are all scalability considerations reported by Milat et al. [1] and Spicer et al. [22]. While Part B focuses on implementation, including domains such as the potential reach, adoption, acceptability of the inetrevention along with potential resource requirements. These domains are consistent with key success factors for scale-up and/or effective implementation identified in the literature [1, 8, 11, 12, 18, 27, 28]. Interestingly, only three [1, 13, 23] of the five existing scalability frameworks and checklists identified in the review covered the concepts of intervention reach and acceptability. These were thought to be vital scalability concepts by respondents in this study and, as such, they feature prominently in the ISAT. The potential adaptability as well as future sustainability of the intervention featured in this section are highlighted in the literature as important considerations in effective scale-up [25, 33–35]. The final section of the ISAT (Part C) provides a summative assessment that highlights the strengths and weaknesses across domains to inform a final decision on whether to scale up an intervention or not. This feature has not appeared in any existing tools identified in the literature. The convergence between the literature, the selected domains and the last round of end-user testing was encouraging, indicating that there were no major omissions identified in the final version of ISAT.

The end users consulted for the study identified a number of important uses for the ISAT, including as a decision support tool, as an advocacy tool, and for use in strategic planning and resource allocation processes. Many of those interviewed noted that political and/or strategic and organisational context and support along with availability of funds, often take priority over evidence of efficacy in scale-up decision-making. However, respondents also believed that a tool like the ISAT would be very useful in bringing to light the evidence base for a particular intervention and in inserting that evidence into decision-making processes through a structured and transparent process. It was noted by respondents that the process of completing the ISAT (especially as a team) provides a structured opportunity through which policy teams can interact with and consider evidence as part of the decision-making process.

We recommend that the tool is completed by a group of stakeholders as it will often be difficult for a single individual to gather evidence for all the domains in isolation. The team assembled to complete the ISAT will be context specific but would generally bring together a range of expertise in research, programme planning, implementation and practice. Teams could involve policy-makers, researchers, implementers and context-specific practice experts. For example, research expertise will facilitate assessments regarding the strength of the evidence, while practitioner/policy-maker experience will be critical in identifying important contextual factors that could affect implementation and the scale-up process.

Utilising a tool like the ISAT as part of the scaling up decision-making process can help policy-makers and practitioners distinguish between effective and implementable interventions. For example, some interventions may not be scalable to the population level despite showing effectiveness in controlled settings due to deficiencies in other important domains such as cost, workforce and sustainability. Completing the ISAT can also enable greater consistency when comparing across interventions competing for the same funds. The process of completing the ISAT itself may also promote learning in how to design programmes for at-scale implementation.

Broadly, the tool has been designed with high-income countries and complex population health interventions in mind as this was identified as a gap in the literature. However, this does not preclude its use in LMICs and other contexts or settings such as human services. The ISAT could also be used by research funding agencies to assess whether they should fund proposals to scale up health interventions and it could be used by researchers in the design of research studies to fill important evidence gaps.

It is important to acknowledge that all the information that would ideally be required to assess the scalability of an intervention may not be available at the time the assessment is made. For example, it might not be possible to accurately determine how large the effect size at a population level needs to be to achieve a population health gain or how much the programme can be changed (to reduce cost or suit different contexts) while still retaining fidelity and outcomes. Where there are gaps in the available evidence, decision-makers may need to consider information from other sources such as expert advice, practice-based knowledge or parallel evidence from similar programmatic interventions in other fields. Where no information is available, a judgement is required about how important the missing information is, whether any gaps can be addressed during implementation, or whether further research is required before scaling up can be recommended. The ISAT provides a systematic way of identifying these gaps and assessing their relative importance.

End users reported that the ISAT could provide guidance on the scalability of efficacious interventions; however, some respondents felt that its ability to provide guidance on the scalability of interventions with weak evidence or those yet to be tested was problematic. We argue that only efficacious/effective interventions should be scaled. Nevertheless, our interviews demonstrated that the reality is often different, with a recent review of scaling up pathways of chronic disease prevention interventions showing that 15% of scaled up programmes identified were based on no discernible evidence of intervention efficacy/effectiveness [36]. In any scale-up process there will likely always be gaps in evidence and, as stated above, it is reasonable to proceed with these gaps in less critical areas. One exception to this rule is intervention efficacy/effectiveness; the risk of scaling up interventions without such evidence is great and may result in the scale-up of programmes that do not work and can divert scarce resources away from potentially effective interventions. This is an important risk that must be managed by decision-makers as it can result in denying or delaying community access to effective services and, ultimately, to the superior health outcomes that these services can provide.

The concepts and content contained in the tool are comprehensive. However, it is not possible for any tool to include every aspect of scale-up nor is this always desirable for practical reasons. It is vital that any tool to be used by decision-makers, like the ISAT, should not be overly onerous to complete. While the tool encompasses elements identified in the literature as key considerations of scalability [1, 11, 13, 18, 22], it is acknowledged that gaps may still exist.

Completing this tool does not negate the need for a comprehensive scale-up plan and/or implementation plan to be developed subsequent to a decision to scale up. Importantly, the information collected by the ISAT could also be used to inform the development of the forthcoming scale-up and/or implementation plan. Further, as some of the end users indicated in their interviews, having the right level of skills mix and staff to complete the tool as a group would be useful but potentially challenging in terms of making it a reality.

### Limitations of the study and further research

This study engaged a small number of scale-up and implementation experts, policy-makers and practitioners, all from Australia. While a larger sample or different respondents may have generated some differing views, the consistency and detail of responses to the semi-structured interviews as well as the substantial experience of the respondents, lend confidence to the external validity of results. Further, the purposeful approach taken in respondent selection, the high response rate (85%) and the comprehensive engagement from all respondents implies that the aforementioned limitations were likely to have been minimised.

The ISAT has been designed to be a practical decision support tool and has been turned into an Australian Prevention Partnership Centre guide for use by policy-makers and practitioners. A logical next step will be to test the usefulness and applicability of the ISAT in real-world intervention policy decision-making processes. We encourage its practical application to policy and programme decision-making in numerous contexts within health and social policy and we intend to conduct further testing of the ISAT in real-world scale-up decision-making processes and to share these as case studies with the field.

## Conclusion

The ISAT fills an important gap in the literature by providing a tool for policy-makers and practitioners to make systematic assessments of the suitability of health interventions for scale-up. The ISAT is designed to stimulate thinking and promote active consideration of the factors that have been shown to be important when assessing scalability. Future research should test the ISAT using real-world case studies. The authors encourage researchers and policy-makers to publish these efforts and to share learnings in the field.

## Supplementary information

**Additional file 1.** Round 1 and 2 Interview Guides.

**Additional file 2.** Intervention Scalability Assessment Tool.

## Data Availability

The datasets used and/or analysed during the current study are available from the corresponding author on reasonable request.

## Abbreviations

ISAT: Intervention Scalability Assessment Tool
LMIC: low- and middle-income country
