# Supplementary material for: Intervention Scalability Assessment Tool: A decision support tool for health policy makers and implementers
Source: Health Res Policy Syst. 2020 Jan 3;18:1. doi: 10.1186/s12961-019-0494-2 (PMC6942323; doi:10.1186/s12961-019-0494-2)
Supplement: Supplementary file 1 — Additional file 1. Round 1 and 2 Interview Guides. [file 12961_2019_494_MOESM1_ESM.docx]

**Supplementary File 1.**

**Round 1 and 2 Interview Guides**

## Round 1 Interview guide

Semi-structured interviews will be conducted to learn more about the scale-up process, and to obtain feedback on the Scalability Assessment Tool. Using a topical guide, interviews will cover four main areas:

1. the participant’s professional background
2. experience in decision-making for scaling up a particular intervention
3. reflections on the scale-up process
4. Feedback on the scalability assessment tool….

To obtain a broad understanding of the scaling-up process at a policy level, we anticipate recruiting participants whose experiences may differ substantially. We will use a topic guide to guide the interview in order to flexibly adapt questions to the particular expertise and experience of each interviewee. The topic guide lays out the overarching topics of interest, and provides sample questions the interviewer may use or adapt to best fit the experience of the participant.

| **Introduction** |
| --- |
| Date for interview:_________________________________________________  Location:_________________________________________________  Respondent(s):___________________________________________  Interviewer:_____________________________________________­­­­­_________  Reporter: ­­­­­­­­­­­­­­­­­­­­­­­­________________________________________________________  *My name is ___ [insert name of interviewer] and this is [insert name of notetaker] and we are both from the University of Sydney. I will conducting this interview and Y will be taking some notes and may have one or two questions later. This is just to make sure we thoroughly capture your experience and opinions.*  *The study investigators have developed a scalability assessment tool to assist people like yourself as well as others with responsibility for scaling up interventions to assess the scalability of a program. It is intended as a companion to the scaling up guide [show guide].*  *We have asked to interview you in particular because of your experience in scaling up of population health interventions. The purpose of this interview is to capture your knowledge and experience on the scale-up process and also your opinion on the tool in terms of its usability and whether it would help in assessing the scalability of a population health intervention.*  *All the information you give to us today will remain strictly confidential. With your permission we would like to record this interview so we have an accurate record of what you have said. The interview will be transcribed and any identifying information either about you or people who you mention will be disguised to preserve anonymity. Obviously there are no right or wrong answers, just your opinion.*  *If you would like to stop the interview at any time for whatever reason, just let me know. You can also withdraw your information in part or in full from the study if you change your mind about participating. The interview should take about 45 minutes to an hour – do you have any time constraints that we should know about before starting? Is there anything you would like to ask me before we get started?*  *Do we have your permission to record the interview? [if yes] Just so we have a formal record of your permission I will now start the recording and ask you again so if you could just respond for the record. Thank-you.*  *Please ensure that you have read the Scalability Assessment Tool before participating in the interview.* |

| **Topic: Professional background** | | |
| --- | --- | --- |
| **Interests** | **Possible questions** | **Objective** |
| Current Position  Previous Experience | 1. What is your current role and how long have you been in your current position? 2. How many years of experience do you have in the development, implementation and or evaluation of population health interventions? 3. Have you ever been involved in decision processes to scale up population health interventions?    1. Frequency, context, role in decision processes, etc.    2. What were the names of the interventions? | Informant’s perspective and experience |

| **Topic:** **Participant’s experience in decision making processes for scaling up population health interventions**  *Ask participant to select a particular intervention to discuss in more detail in this section of the interview. If the participant has had a chance to try the SAT, discuss the intervention used during the trial.* | | |
| --- | --- | --- |
| **Interests** | **Possible questions** | **Objective** |
| Context | 1. Describe the Intervention 2. Describe the need for the intervention/broader context 3. Why was the intervention considered for scale up?   PROMPT   - resources available - political climate - evidence - alignment with strategic priorities | Describe background information and context for scale up |
| Processes | 1. Describe any formal processes that were undertaken in order to make decisions about scaling up the intervention 2. Who was involved in these decision processes? 3. What was your role in the process? | Describe the process of decision making |
| Identification and selection of interventions to scale | 1. How were interventions for scale-up identified? What process was used? 2. Were multiple interventions identified?    1. If so, how was one selected? What was this process like? What were the relevant considerations? 3. How was an intervention chosen in the end?    1. What were the key considerations? | Examine how interventions were selected and compared |
| Role of Evidence | 1. What was the role of evidence in the decision-making process?    1. What kinds of evidence was used (research evidence? Other sources? Expert opinion?)    2. How was evidence used? What decisions did it inform? What evidence was lacking? 2. What information sources were most/least useful? | Explore the role of evidence in decision making |
| Outcomes | 1. What barriers/facilitators to decision-making were encountered?    1. How did these influence the process? 2. What were the final outcomes? 3. How did the decision-making process compare to other kinds of decision-making processes? 4. Were you satisfied with the process?    1. How could it be improved? | Reflect on the decision-making process and outcomes |
| **Topic: Reflection on the process of scaling up interventions** | | |
| **Interests** | **Possible questions** | **Objective** |
| Role of stakeholders | 1. From your experience, what roles do policy makers and researchers play in the process of scaling up population health interventions? | Understand the key stakeholders and their roles in scaling up |
| Influences on decision making | 1. From your experience, what are the most powerful influences on decisions to scale up population health interventions? 2. What should they be?    1. If what is differs than what should be, why is this so? What could be done to correct it? | Examine other influences; compare and contrast the ideal with the status quo |
| Influences on scaling up  (if participant has had previous experience implementing scale up) | 1. In your opinion, what are the key barriers to scaling up interventions?    1. What, if anything, would ameliorate these barriers? 2. What are the key considerations when taking a program to scale? 3. How did the strategic context influence scale up? 4. What kind of information would be most useful to you if undertaking scale up? | Explore influences on executing scale up efforts |

| **Topic: Feedback on the Scalability Assessment Tool**  **Now thinking specifically about the draft Scalability Assessment Tool.**  The Scalability Assessment Tool (SAT) is designed to be used by health practitioners, policy makers and others with responsibility for scaling up health interventions across health systems. Though developed for use in health contexts the SAT could also be used in other human service contexts. Specifically the SAT is designed to:   - assess of suitability of an intervention/s for scale up; - assess contextual factors that are potential facilitators or barriers to scale up; - identify information and evidence gaps; and - identify ways to improve intervention scalability | | |
| --- | --- | --- |
| **Interests** | **Possible questions** | **Objective** |
| Feedback on the purpose and targeted users of the tool | 1. Do you have any comments/suggestions about the goal of the tool? 2. The key target audiences for the tool are policy makers, practitioners with responsibility for determining whether to scale up population health interventions. Is this tool suitable for these or other audiences? 3. Is a tool with these aims needed? | Examine appropriateness of the SAT tool |
| Feedback on the design of the tool | 1. What was the process of completing the tool like? 2. Comments on the structure/design & presentation of the tool? | Consider whether the SAT design is useful |
| Feedback on the applicability of the tool | 1. What did you learn from using the tool? 2. What are the key themes presented? Are some themes more or less relevant than others?    1. Are there any missing themes? 3. How would you envision using the tool? 4. Have you used tools like these before? Why or why not? What qualities are or are not helpful in a tool? | Examine the relevance of the SAT |
| **Topic: Closing** | | |
| **Interests** | **Ideas for questions** | **Objective** |
| Opportunity for other feedback | 1. Is there anything you’d like to share that we didn’t cover in this conversation?    1. Particularly as it relates to understanding scaling up or using tools in policy contexts | Provide opportunity to catch something otherwise missed over course of interview |

## Round 2 Interview guide

Semi-structured interviews will be conducted to learn more about scale-up processes, and to obtain feedback on a new developed Scalability Assessment Tool. Using an interview guide, interviews will cover four main areas:

1. the participant’s professional background
2. experience in decision-making for scaling up a particular intervention
3. general reflection on the scale-up process
4. feedback on the scalability assessment tool….

To obtain a broad understanding of the scaling-up process at a policy level, we anticipate recruiting participants whose experiences of scaling up interventions may differ substantially. We will use this guide to structure the interview. The guide allows for the adaptation of questions to the particular expertise and experience of interviewees. The guide lays out the overarching topics of interest, and provides sample questions the interviewer may use or adapt to best fit the experience of the participant.

| **Introduction** |
| --- |
| Date for interview:_________________________________________________  Location:_________________________________________________  Respondent(s):___________________________________________  Interviewer:_____________________________________________­­­­­_________  Reporter: ­­­­­­­­­­­­­­­­­­­­­­­­________________________________________________________  *My name is ___ [insert name of interviewer] and this is [insert name of notetaker] and we are both from the University of Sydney. I will be conducting this interview and Y will be taking notes and may have one or two questions later.*  *The study investigators have developed the Scalability Assessment Tool (SAT), which was sent to you earlier, to assist people like yourself as well as others to assess the scalability of a program. It is intended as a companion to the scaling up guide [show guide].*  *You have been selected for the interview because of your experience of the scale-up of population health interventions. We would firstly, like to get your reflections on the scaling up process and the useability and utility of the SAT tool.*  *All the information you give to us today will remain strictly confidential. With your permission we would like to record the interview so we have an accurate record of what you have said. The interview will be transcribed and any identifying information either about you or people who you mention will be disguised to preserve anonymity.*  *Obviously there are no right or wrong answers to the questions - just your opinion. If you would like to stop the interview at any time for whatever reason, just let us know. You can also withdraw your information in part or in full from the study if you change your mind about participating.*  *Please ensure that you have read the Scalability Assessment Tool before participating in the interview. The interview should take no longer than an hour - do you have any time constraints that we should know about before starting?*  *Is there anything you would like to ask me before we get started?*  *Do we have your permission to record the interview? [if yes] Just so we have a formal record of your permission I will now start the recording and ask you again so if you could just respond for the record. Thank-you.* |

| **Topic: Professional background** | | |
| --- | --- | --- |
| **Interests** | **Possible questions** | **Objective** |
| Current Position  Previous Experience | - Firstly, I just wanted to get some information about yourself and your background. - Can you please describe your current role and your experience in scaling up interventions and the role you played in those? - What were some of the interventions that you have had experience in scaling up and what role did you play in those?  1. What is your role and how long have you been in your current position? 2. How many years of experience do you have in the development, implementation and or evaluation of health interventions? 3. What experience do you have of the scaling up of interventions?   PROMPT   - As a decision maker - As an implementer - As a planner  1. What were the names of the interventions? | Informant’s perspective and experience |
| **Topic:** **Participant’s experience in a decision making process to scale up a population health intervention**  *Ask the participant to* ***select a particular intervention*** *to discuss in detail in this section of the interview.* | | |
| **Interests** | **Possible questions** | **Objective** |
| Context | - Now, just wanting to unpack the process of scaling up a little more, from your experience, is there one intervention/program that has been scaled up in particular that you could describe the process in more detail? - Could we start by, firstly, describing the intervention and identifying what the need was for that intervention within the broader context? - So, why then was the intervention considered for scale up?   PROMPT   - resources available - political climate - evidence - alignment with strategic priorities | Describe background information and context for scale up |
| Processes | - Can you describe what processes were undertaken in order to make the decision to scale up the intervention?   PROMPT   - Who was involved in these processes? - What was your role in these processes? | Describe the process of decision making |

| **Topic: General reflection on the process of scaling up interventions** | | |
| --- | --- | --- |
| **Interests** | **Possible questions** | **Objective** |
| Role of stakeholders | - I would now like just to get some general reflections on the process of scaling up interventions… In your experience, what roles do key stakeholders play in the process of scaling up population health interventions?   PROMPT   - Policy makers? - Researchers? - NGOs? - The community? - Others? | Understand the key stakeholders and their roles in scaling up |
| Identification and selection of interventions to scale up | 1. In your experience, how are interventions for scale-up identified?   PROMPT   - What processes are used?  1. When multiple interventions are identified:  - How is one selected? - What is the selection process like? - What are the relevant considerations? | Examine how interventions are selected and compared |
| Role of Evidence | 1. In your experience, what is the role of evidence in the decision-making process?   PROMPT   - What kinds of evidence are used? (e.g. research evidence, expert opinion or other types of evidence) - How is evidence used? - What decisions does it inform? - What evidence is generally lacking that could inform a decision? | Explore the role of evidence in decision making |
| Influences on decision making | 1. **In your experience, what are the most powerful influences on decisions to scale up population health interventions?** 2. What should they be? 3. If what is differs to what should be:  - Why is this so? - What could be done to correct it? | Examine other influences; compare and contrast the ideal with the status quo |
| Intervention adaption | 1. Now just going back to the intervention we discussed before…once a decision was made to scale it up was the intervention adapted in any way?   If yes, how and why was it adapted?  PROMPT   - Modification of aims and objectives - Target groups - Engagement of stakeholders - Intervention governance - Funding and resources - Implementation - Evaluation | Examine adaption of interventions and why adaption was required |
| Influences on scaling up  (if participant has had previous experience implementing scale up) | 1. In your experience, what are the key barriers to scaling up interventions?  - What, if anything, would ameliorate these barriers?  1. What are the key considerations when scaling up? 2. How does the broader policy context influence scaling up? 3. What kind of information would be most useful to you if considering scaling up? | Explore influences on executing scale up efforts |

| **Topic: Feedback on the Scalability Assessment Tool**  **Now thinking specifically about the draft Scalability Assessment Tool.**  The Scalability Assessment Tool (SAT) is designed to be used by health practitioners, policy makers and others with responsibility for scaling up health interventions across health systems. Though developed for use in health contexts the SAT could also be used in other human service contexts.  Specifically the aims of the SAT are to:   - assess of suitability of an intervention/s for scale up; - assess contextual factors that are potential facilitators or barriers to scale up; - identify information and evidence gaps; and - identify ways to improve intervention scalability | | |
| --- | --- | --- |
| **Interests** | **Possible questions** | **Objective** |
| Feedback on the purpose and targeted users of the tool | 1. Do you have any comments/suggestions about the aims of the tool?   *The key target audiences for the tool are policy makers, practitioners with responsibility for determining whether to scale up population health interventions.*   1. Is the tool suitable for these and other audiences? 2. Do you think a tool such as the SAT would be useful in your work? | Examine appropriateness of the SAT tool |
| Feedback on the design of the tool | 1. What was the process of completing the tool like? 2. Please provide comments on the structure/design & presentation of the tool? | Consider whether the SAT design is useful |
| Feedback on the applicability of the tool | 1. What did you learn from using the tool? 2. Are some sections more relevant than others?   PROMPT   - Are there any components missing?  1. How would you envisage using the tool? 2. Have you used tools like these before?   PROMPT   - Why or why not?  1. What qualities are helpful or not helpful in such a tool? | Examine the relevance of the SAT |
| **Topic: Closing** | | |
| **Interests** | **Ideas for questions** | **Objective** |
| Opportunity for other feedback | 1. Is there anything you’d like to share that we didn’t cover in this conversation?   PROMPT   - For example, understanding scaling up or using tools in policy contexts | Provide opportunity to catch something otherwise missed over course of interview |
